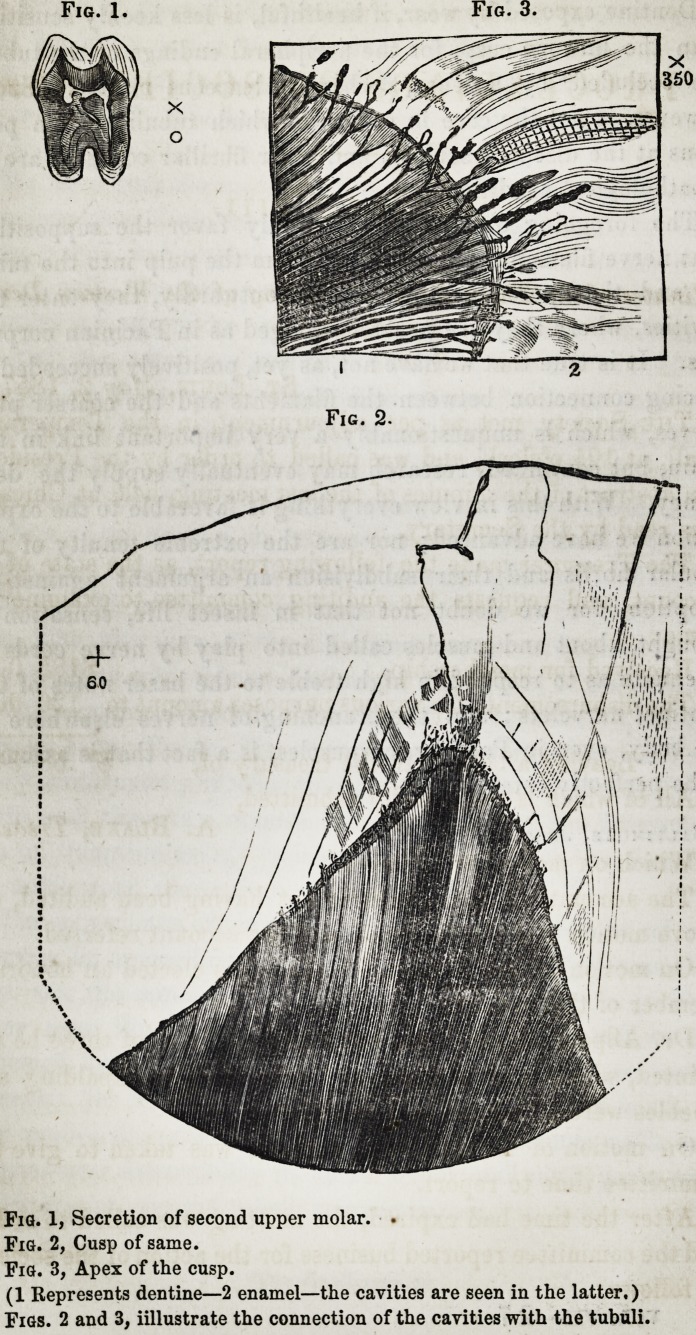# Contribution to the Minute Anatomy of the Teeth

**Published:** 1857-07

**Authors:** Christopher Johnston

**Affiliations:** Lecturer in the Baltimore College of Dental Surgery.


### ARTICLE VII.

*Contribution to the Minute Anatomy of the Teeth.* By CHRISTOPHER JOHNSTON, M. D., Lecturer in the Baltimore College of Dental Surgery.

IT has hitherto been a matter of astonishment that organs of such extreme hardness as the teeth should manifest, both normally and pathologically, a very delicate sensibility, while the whole thickness of the dental tissues was supposed to separate the surface recipient of impressions from the nerve bearing pulp in health, and a considerable, but of course, variable amount of tooth substance in states of disease. The exaltation of general sensibility to an exquisite degree in morbid conditions was not, however, the most surprising circumstance in connection with the "animation" of the teeth, but the ready recognition of the smallest particles, their quality and situation, especially by the masticating surface, and also the impressions created by chemical substances. To explain several of these phenomena it was assumed that vibrations of the hard tissues rouse the pulp nerves to perception; or else that in caries, compression of fluid into the dentinal canaliculi finally exerted its influence upon the pulp itself.

The attentive consideration that we have given to the dental structures leads us to believe that some important points have escaped the notice of other observers, among which may be mentioned the extent and manner of nervous distribution in a tooth, and the ultimate anatomical constitution of the dentine: and these matters form the subject of the present paper.

If a fine longitudinal section of a perfect human tooth be viewed under the microscope, we may observe that differences exist between the tubuli of the crown and of the fang, not only as regards their general course but also with respect to the size and character of their terminations. In the crown their direction is nearly straight from the pulp to the point of each cusp, on either side of which they bend slightly so as to arrive at the surface of the dentine in lines nearly perpendicular to it; then, next the pulp, the "primary curvatures" makes their appearance, the first concavity being upwards and followed outwardly by a convexity of great extent and long radius, and a slight concavity marks their ending: in the fang the tubuli, as we recede from the neck, approach horizontality more and more, without, however, reaching it, the bending becomes less conspicuous, and the inner curvature is the greatest.

The tubuli of the crown sometimes divide dichotomously and send off here and there a communicating branch—they are also best with exceedingly fine ramuscules, the attenuated extremities of which are lost in the intertubular space; and although they taper gradually in approaching the enamel, they reach the surface of the dentine either in a single coarse trunk or else by two, or more rarely three branches of considerable diameter. But although in ill made specimens the tubuli may be traced no farther, we have assured ourselves that each and every one of these terminal extremities or branches penetrates into the enamel and communicates—at times singly—with a remarkable elongated cavity. The cavities thus imbedded in the enamel are irregular, present constrictions and are oftentimes not straight: at the apex of each cusp they are thickly set and may even attain a length of 0.095 millimetre with an average breadth of nearly 0.006 millimetre, and they extend from lesser cusps which distinguish the outer and inner surfaces of the anterior teeth, especially the incisors. But they diminish in frequence and length until finally they disappear at the coronal margin of the enamel. Of course in no specimen do the cavities correspond in number with the tubuli visible, for both do not often lie in the plane of the section, and besides two or more tubuli may end in the same dilatation, or else abruptly divided tubes may be noticed having been superated by the process of preparation from the remaining portion of the cavity to which they belonged.

We are aware that these interesting dilatations have been previously pointed out,* but so far from regarding them as

* Koelliker, Mikroscop. Anat. &c.

"pathological" we are constrained to consider them as normal and constant, and, therefore, consequential. Neither are they nor ought they to be peculiar to humanity; comparative study discovers them in the teeth of the horse, finds them particularly evident in the elephant, and also with varying distincness in hippopotamus, mastodon, elephas primigenius, in sheep, moose deer, mouse, hare, squirrel, common bat and alligator. In the only marsupial examined by us, the Virginia opossum,* we have detected the cavities in the enamel lying upon the outer surface of the dentine of the crown; but what is more surprising, the dentinal tubuli, in all that part of the tooth, transverse the enamel almost as far as its surface, being continued beyond the cavities in situations where these exist.

No such appearances manifest themselves in the fang; but the tubuli give off lateral branches more or less profuse and distinct, primary subdivisions are more frequent than in the crown, and the canals, constantly ramifying and inosculating, terminate in exquisitely delicate ramuscles, some of which may be followed into the irregular space of Tomes' intermediate layer, and some are seen to communicate with twigs from the cemental lacunæ.

We have not recognized either canals or spaces in the enamel, other than those described above; nor have we met with tubular prolongations through that substance in any other mammal except the opossum; but this arrangement appears to obtain in fishes, as may be verified in Belone, (silver gar.) We beg leave here to interpolate the observation, that the serrations upon the enamel columns, intimately associated with the transverse striation, tend largely, by their interlocking, to fortify the whole structure; and that this *striation*, with all deference to the learned author of Odontography, is certainly present in the enamel of elephas indicus, and elephas primigenius, while it is so evident in that of mastodon giganteus, as strongly to recall human muscular fibre.

If we now turn from dry specimens, or those mounted in

* Didelphys Virgin.

balsam, to wet preparations, we shall find the study both interesting and profitable.

Make a tolerably thin longitudinal section through the cusps of a perfect human tooth, (a bicuspid or second upper molar is the easiest to obtain unworn,) by grinding with water upon a fine corundum wheel, and subsequently on a good Arkansas hone, and after washing, inspect it under a low power and notice the character and dimensions of the cavities in the enamel already mentioned, especially around the points of the dentine. Next prepare a trough by sticking bands of white wax upon a common slide, deposit the section in the enclosure and cover it with water. If we now withdraw the remaining pulp, making traction towards the root, that whole margin of that body, particularly the apicial portion, appears shaggy with minute fibrils that were unquestionably lodged in the beginnings of the tubuli.* Remove the pulp and the water, place a fragment of a slide upon the fang of the section, and overflow it with very dilute chlorhydric acid. Bubbles instantly arise from the enamel, nevertheless, we can perceive that the columns and the striæ become more conspicuous before they disappear; and finally, when the enamel is entirely dissolved we will find many of the cavities represented by transparent bodies of the same or similar form attached to the dentine, by prolongations of the tubuli. Now carefully perforate the wax wall, and draw off the fluid, detach the wax and lay it aside, cover the specimen with a large thin cover-glass, and add pure acid at the edge of the latter until it fills up the space between the two glasses. Presently the margins of the dentine begin to clear, and the intertubular substance is no longer homogeneous, but exhibits transverse (to the tubuli) striations similar to those of enamel, and which, indeed, is sometimes perceptible in sections mounted in balsam, caused by juxtaposed molecules, that, according to our measurements, do not exceed 0.0024 millimetre. This, which we hold to be a demonstration of the ultimate constitution of dentine, passes quickly away, unless we choose to arrest

* Tomes previously.

the action of the acid by plunging the specimen in dilute alcohol; but if its action be suffered to continue for a day or two, nothing will remain but the tubuli perfectly isolated in their whole length.* We have found it impossible to retain the cavity bodies.

We have thus shown, in confirmation of Tomes, that organic filaments pass from the pulp into the dentinal tubuli; (2) that these tubuli, and probably the remaining portion of the contained pulp filament may be completely isolated by the destructive agency of an acid upon the intertubular substance; (3) that the tubuli are normally continued into the moliniform cavities in the enamel which characterize its inner stratum; (4) that the tube-substance fills these cavities, since the same reagent which spares the tubuli of the dentine, respects the organic matter of the "cavity bodies," and leaves them, after solution of the enamel, in connection with the tubuli; (5) and lastly, that intertubular dentine is an aggregation of molecules, about 0.0024 millimitres in diameter. Here be it noted, however, that this view of the ultimate structure of dentine in no wise conflicts with demonstrations by M. Salter, of the *manner* of development of dentine, or rather of its invasion by the calcareous salts; for in this matter our own observations are entirely confirmatory of what that gentleman has so strikingly set forth, and, therefore, oppose our acceptance of the interpretation of "dentine cells," "*zwischenräume*," &c., to be found in the noble work above cited.

The experience of enlightened dental practitioners of our country, in operations upon the teeth—and we presume that teeth are the same everywhere—is, that sensibility is greatest at or near the party line of dentine and enamel; and we are informed by Prof. Maynard, of Washington, that in preparing a cavity for filling, particularly upon the side of a tooth, he first applies the excavator to the inferior and inner walls, and so produces insensibility in those upper and outer parts with which the intercepted tubuli communicated.

* As also Koelliker.

Dentine exposed by wear, if healthful, is less keenly sensitive than the unworn cusp, for the peripheral endings of the tubuli are occluded* by a dense, perhaps calcareous matter. Such, however, is not the case in caries, in which tubuli remain pervious at the diseased surface, and their fibrillar contents are in a pathological condition.

The foregoing observations certainly favor the supposition that nerve filaments are continued from the pulp into the tubuli; and that pursuing their course outwardly, they enter the cavities, where they are probably lodged as in Pacinian corpuscles. It is true that we have not, as yet, positively succeeded in tracing connection between the filaments and the coarser pulp nerves, which is unquestionably a very important link in the chain, but continued research may eventually supply the deficiency. With this in view everything is favorable to the explanation we have advanced; nor are the extreme tenuity of the tubular fibrils and their subdivision an argument against its adoption, for we doubt not that in insect life, sensation is brought about and muscles called into play by nerve cords so attenuate as to respond in high treble to the baser notes of the dentinal nervelets; and the branching of nerves elsewhere in the body, even in Pacinian corpuscles, is a fact that is assumed to be perfectly established.

BALTIMORE, *June 1st*, 1857.

* Tomes and others.

FIG. 1.

FIG. 3.

FIG. 2.

FIG. 1, Secretion of second upper molar.
FIG. 2, Cusp of same.
FIG. 3, Apex of the cusp.
(1 Represents dentine—2 enamel—the cavities are seen in the latter.)
FIGS. 2 and 3, iillustrate the connection of the cavities with the tubuli.

## Figures and Tables

**Figure f1:**